# Dedifferentiated chondrosarcoma of the cervical spine: a case report

**DOI:** 10.1186/1477-7819-11-32

**Published:** 2013-02-02

**Authors:** Yoshihiro Matsumoto, Yusuke Takahashi, Katsumi Harimaya, Takeshi Nakagawa, Kenichi Kawaguchi, Seiji Okada, Mitsumasa Hayashida, Toshio Doi, Akio Sakamoto, Tomoya Matsunobu, Yoshinao Oda, Yukihide Iwamoto

**Affiliations:** 1 Department of Orthopedic Surgery, Graduate School of Medical Sciences, Kyushu University, 3-1-1 Maidashi, Higashi-ku, Fukuoka 812-8582, Japan; 2 Department of Anatomic Pathology, Graduate School of Medical Sciences, Kyushu University, 3-1-1 Maidashi, Higashi-ku, Fukuoka, 812-8582, Japan; 3 Department of Orthopedic Surgery, Kyushu University Beppu Hospital, 3-1-1 Maidashi, Higashi-ku, Fukuoka, 812-8582, Japan

**Keywords:** Dedifferentiated chondrosarcoma, Cervical spine, Histology

## Abstract

Dedifferentiated chondrosarcoma (DDCS) is a rare and aggressive bone tumor with poor prognosis. Primary DDCS of the mobile spine is extremely rare, particularly in the cervical spine. We herein describe a first case of cervical DDCS in an 81-year-old male presenting with a slowly growing mass. Radiographs showed an expansion of the cortical contour of the C2 lamina and a soft tissue mass with punctate calcification. Magnetic resonance imaging demonstrated a lobulated lesion expanding over the entire lamina and pedicles of C2 with the tumor protuberant to the adjacent soft tissue. A complete tumor resection was performed. Histologically, the majority of the tumor was a low-grade chondrosarcoma component. However, atypical spindle cells that had proliferated in a fascicular pattern with a collagenous stroma, mimicking fibrosarcoma, were focally observed without a transitional zone, and these features confirmed that the tumor was DDCS.

## Background

Primary chondrosarcoma (CS) is the third most common primary malignant tumor of bone [1]. There are several classifications of CS. One is based on localization, namely, central, peripheral and periosteal. Another one is based on histology and by far the most common histological subtype is conventional CS. Additionally, there are also some rare histological entities, clear-cell CS, mesenchymal CS, and dedifferentiated CS (DDCS). Despite the continuous development and progress made in diagnostic techniques and auxiliary treatment, there is still no good treatment for DDCS at present. The prognosis of the patients of DDCS remains poor and the five-year survival rate of DDCS ranges around 24% in the most recent European series [2,3]. Because of its rarity, only a few large studies regarding the demographic data of DDCS have been reported. In one of these large series, Grimer *et al*. [2] showed that almost two-third of the cases were located in long bones of the peripheral skeleton, mainly the femur, while the remaining one-third involved the axial skeleton. However, DDCS arising in the mobile spine was unusual. To our knowledge, this is the first case report of cervical DDCS that provides the detailed radiological and histological findings along with a review of the literature.

## Case presentation

**Figure 1:**
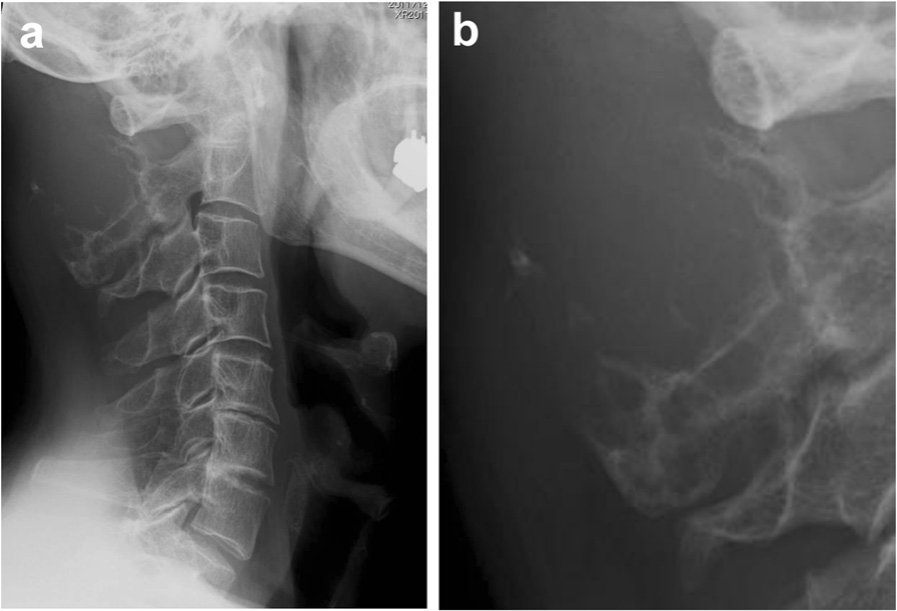
**Plain radiographs of the cervical spine ((a) lateral view, (b) zoomed lateral view of the C2 spinous process) showed an expansion of the cortical contour of the C2 lamina and a soft-tissue mass with punctate calcification. **Scalloping of the spinous process of C3 was also observed.

**Figure 2:**
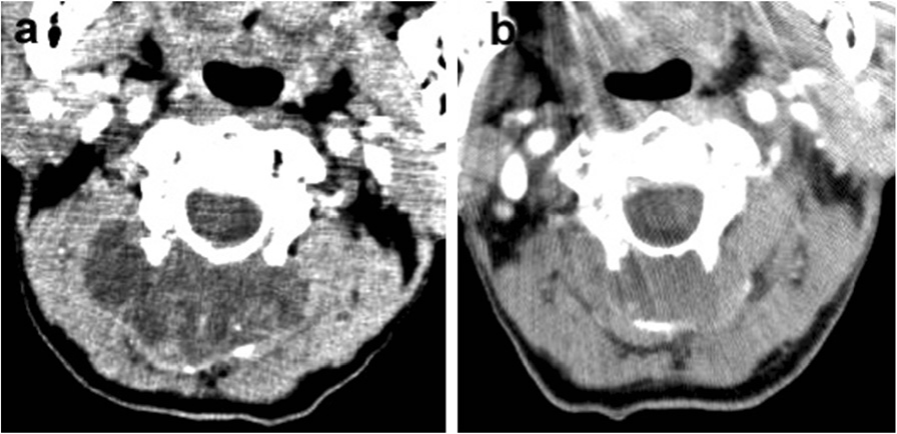
**The axial computed tomography (CT) image (a) demonstrated a soft-tissue mass with an amorphous ‘rings and arcs’ calcified matrix and destruction of the C2 lamina. **The tumor at the C2 lamina was also observed in a CT image taken at two years before presentation (**b**) and the tumor was noted to have expanded during the follow up.

**Figure 3:**
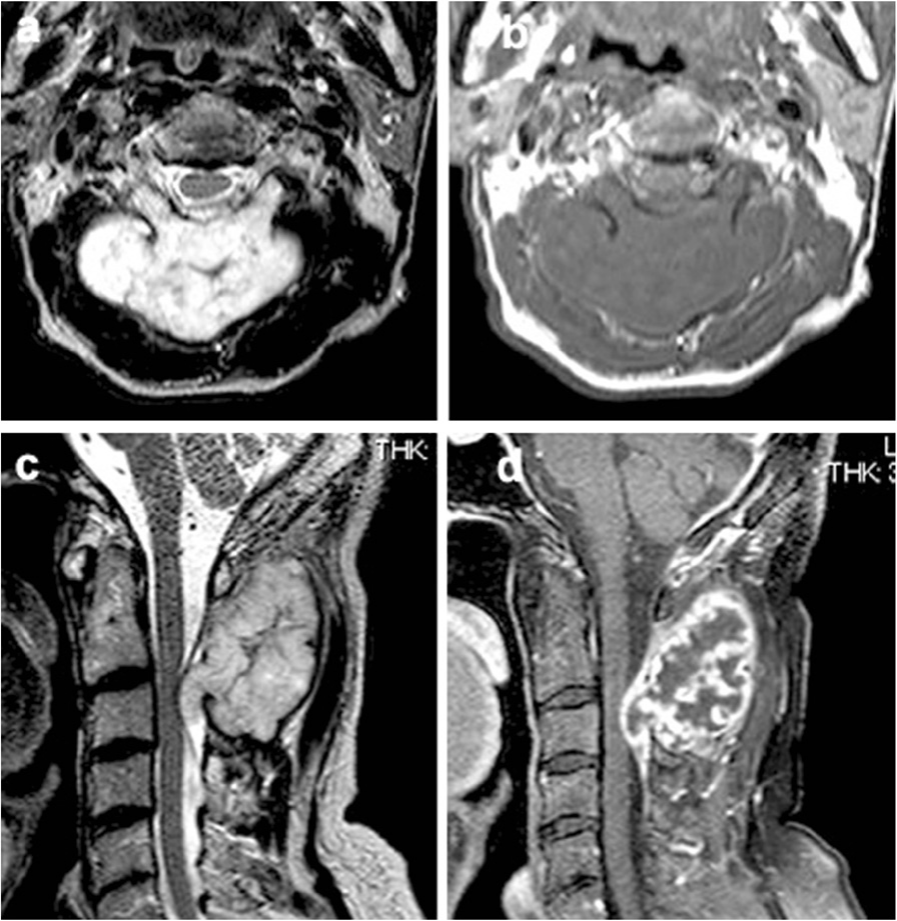
**Transverse T2 (a) and T1-weighted images at C2 (b), a sagittal T2-weighted image (c) and T1-weighted image after the intravenous administration of gadolinium (d), of the cervical spine. **During the MR imaging, the lesion exhibited a lobulated lesion expanding over the entire lamina and pedicles of C2 with the tumor protuberant to the adjacent soft tissue ((**a**) and (**b**)). The mass was low-intensity on T1-weighted images and had heterogeneous low and high-intensities on T2-weighted images, suggesting the presence of mineralized and nonmineralized matrices. Gadolinium-enhanced T1-weighted fat saturated images showed intense peripheral and lobulated rim enhancement, whereas lesions with limited mineralization may appear with homogenous enhancement (**d**). MRI also showed the epidural extension of the mass at the C2/3 level (**c**).

An 81-year-old male consulted our department with a six-month history of swelling and mild cervical spinal stiffness. He also noticed a slowly growing mass. He had a history of malignant lymphoma that was treated by multi-agent chemotherapy and had achieved a partial remission. His physical examination was unremarkable except for the presence of a hard upper cervical spine mass. The clinical blood examination did not show any abnormality. Radiographs of the cervical spine showed an expansion of the cortical contour of the C2 lamina and a soft tissue mass with punctate calcification. Scalloping of the spinous process of C3 was also observed (Figures 1a and b). The axial computed tomography (CT) image (Figure 2a) demonstrated a soft-tissue mass with an amorphous ‘rings and arcs’ calcified matrix and destruction of the entire C2 lamina. Importantly, these features were also present in a CT image taken two years before the presentation (Figure 2b) showing the tumor had expanded during the interim period. Magnetic resonance imaging (MRI) showed a lobulated lesion expanding over the entire lamina and pedicles of C2 with the tumor protuberant to the adjacent soft tissue. The mass was low-intensity on T1-weighted imaging and had heterogeneous low and high-intensities on T2-weighted imaging, thus suggesting mineralized and nonmineralized matrices. (Figures 3a and b) Gadolinium enhanced T1-weighted fat saturated images showed intense peripheral and lobulated rim enhancement, whereas lesions with limited mineralization may appear with homogenous enhancement (Figure 3d). MRI showed the epidural extension of the mass at the C2/3 level (Figure 3c). All of these imaging findings, along with the clinical presentation, suggested the chondrogenic nature of the mass, particularly suggesting a low-grade CS. An open biopsy showed multilobular proliferation of chondrocytic cells with mild cellular atypia embedded in abundant chondroid matrix, suggesting a grade I CS. A CT examination of the chest, abdomen, and pelvis was normal. A complete intralesional resection of the tumor was performed by the posterior approach, with instrumentation being placed from C1 to C4 and fusion was then accomplished by an autograft made with bone chips. Macroscopically, the tumor was completely removed. The wound was extensively irrigated and closed in multiple layers. There were no neurological deficits and no wound problems were observed postoperatively.

**Figure 4:**
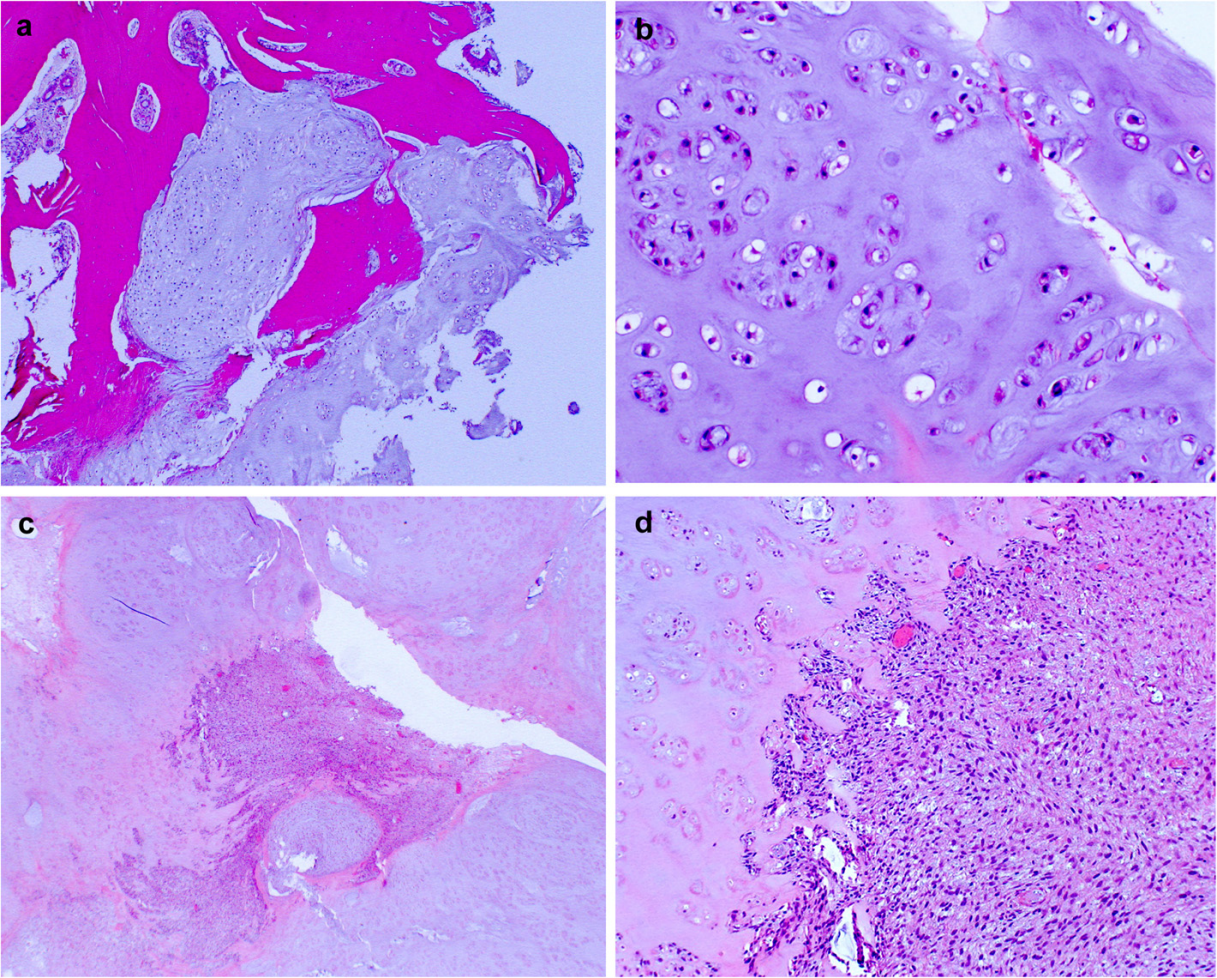
**Hematoxylin and eosin staining of surgical specimens: (a) The cartilaginous component was permeating into the normal trabecular bone (x40) and showed well-differentiated conventional chondrosarcoma composed of atypical chondrocytes with hyaline cartilage matrix (b) (x200). (c) **(x40) and (**d**) (x100) Characteristic coexistence of conventional chondrosarcoma and dedifferentiated components. The dedifferentiated components were composed of atypical spindle cells that had proliferated in a fascicular pattern with collagenous stroma resembling fibrosarcoma.

**Figure 5:**
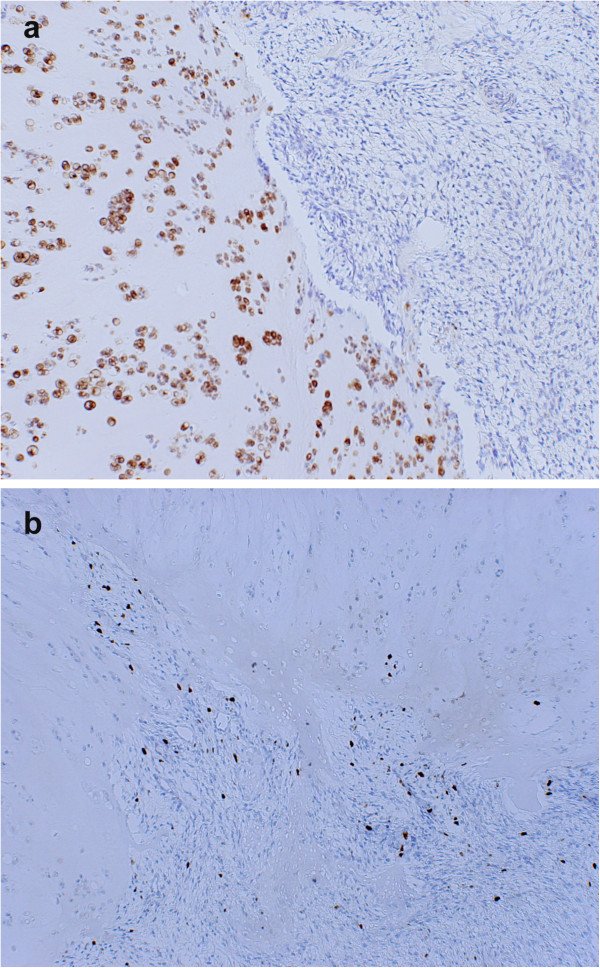
**In the immunohistochemical analysis, the cartilaginous components were positive for S-100 protein, but the spindle cell areas were negative (a) (x100). **(bcp (x100) Ki-67 antigen, a marker of cell proliferation, was increased in the spindle cell areas, while it was absent in the cartilaginous component.

Microscopically, the majority of the tumor was composed of the proliferation of chondrocytic cells with enlarged hyperchromatic nuclei, accompanied by chondromyxoid matrix and permeating bone trabeculae indicating low-grade CS (Figures 4a and b). The low-grade (grade I) CS component was demarcated to the atypical spindle cell areas (Figure 4c). Atypical spindle cells proliferated in a fascicular pattern with collagenous stroma mimicking fibrosarcoma (Figure 4d), thus indicating it to be fibrosarcomatous dedifferentiation of CS. The extension of high-grade component was around 10%. Together, these findings led to a final diagnosis of DDCS because of the coexistence of conventional low-grade CS and dedifferentiated components with sharp margins. In an immunohistochemical analysis, the cartilaginous components were positive for S-100 protein, but the spindle cell areas were negative (Figure 5a). The Ki-67 antigen, a marker of cell proliferation, was increased in the spindle cell areas, but not in the cartilaginous component (Figure 5b). A postoperative MRI and a CT scan performed four months after the operation showed no evidence of local recurrence or distant metastasis. However, six months after the surgery, the patient died from rupture of the colon due to the recurrence of malignant lymphoma.

## Discussion

The term DDCS was introduced in 1971 [4]. It is thought that up to 11% of low-grade CS contain an additional component of high-grade mesenchymal sarcoma features, including conventional osteosarcoma, malignant fibrous histiocytoma, fibrosarcoma or anaplastic spindle-cell sarcoma [5]. These dedifferentiated components are thought to be related to the characteristic clinical features of DDCS, such as its locally aggressive behavior, frequency of recurrence and, poor prognosis. DDCS usually affects long bones such as the proximal femur, humerus and tibia. DDCS in the spine is extremely rare, and to the best of our knowledge, this is the first case of DDCS in the cervical spine reported in detail. The clinical course of low-grade CS originating in the spine is usually long because of the slow growth of the tumor [6]. However, treatment of DDCS in the spine would be complex and challenging because of its clinical features as discussed above.

In the 57 cases of DDCS reported, calcification was found in around 50% of the lesions and an extraosseous mass was observed in roughly 55% of tumors [5]. CT is able to clearly demonstrate these features as well the anatomic origin of the lesion [7]. In this case, the origin of the tumor was thought to be the C2 lamina, which was confirmed by successive CT images (Figures 2a and b). MRI demonstrates the tumor as a low-signal intensity region on T1-weighted images and as heterogeneous low and high-signal intensities on T2-weighted images, thus suggesting the presence of mineralized and nonmineralized matrices. Fat suppressed contrast-enhanced T1-weighted images show peripheral and lobulated rim enhancement, whereas lesions with limited calcification may appear with homogenous enhancement, know as ‘rings and arcs pattern’ [8]. Notably, MRI is superior to CT in terms of describing the epidural and intraforaminal extension [9], as was seen in this case. Even if the biopsy results show low-grade CS, excluding a diagnosis of high-grade chondrosarcoma, clinicians should consider the possibility of DDCS in patients with destructive bone lesions and prominent soft tissue masses.

CS, particularly DDCS, is known to be resistant to the chemotherapy and radiotherapy. Thus, surgery remains the only potentially curative treatment for DDCS. The local recurrence rate of DDCS rate was over 50% in previous studies, but this decreased after radical surgery with a clean surgical margin [10,11]. Regarding the treatment of spinal tumor, *en bloc* resection with negative margins has been shown to decrease the rates of local and metastatic recurrence [12,13]. Therefore, an *en bloc* resection, if possible, is now recommended for many primary tumors of the thoracic, lumbar, and sacral spine. However, in cases affecting the cervical spine, the proximity of the vertebral arteries, the complex bony structure, and the importance of the cervical nerve roots prevent the use of an ideal *en bloc* resection [14]. In the present case, total *en bloc* resection of the tumor was practically impossible because of the bilateral tumor invasion into the C2 pedicle, so tumor curettage was performed. Although the tumor was totally removed macroscopically, the recurrence of the tumor was thought to be inevitable and a careful postoperative observation would be needed in such case.

As mentioned above, DDCS is not sensitive to radiotherapy. However, if the tumor cannot be completely resected, or in cases of local recurrence, radiotherapy should be considered. Conventional radiotherapy using spinal cord dose limits of 45 to 50 Gy, given to the full cross section of the cord, is well below the dose needed to control most sarcomas including CS [15]. Therefore, innovative modalities of radiotherapy are necessary to safely irradiate these tumors with an intension to cure. In this regard, proton/photon beam radiation is promising because of its excellent physical dose distribution and intensity modulation, which largely spares the spinal cord. A pilot study of patients with axial skeleton tumors treated with combined high-dose proton/photon radiation showed satisfactory rates of local control (LC). For CS, LC was achieved in six out of six patients with a mean target dose of 73.9 gray equivalent (GyE), although the histological subtype of CS was not specified in that study [16]. The use of heavier charged particles, in particular carbon ions, would be another interesting alternative. Kamada *et al*. [17] reported the results of a Phase I/II study of carbon- ion RT in 57 patients with 64 sites of unresectable sarcomas of bone and soft tissue and a total of 19 patients with spinal or paraspinal tumors were enrolled. The total dose given was 52.8 to 73.6 carbon GyE. In this study, the LC rates for CS were 71% (5 out of 7 patients) without unacceptable morbidity. These results suggest that proton/photon radiation and carbon-ion RT may provide an effective, safe local treatment for patients with cervical spine CS [17].

Distant metastasis, particularly lung metastasis, is the main cause of death due to DDCS and the development of effective adjuvant chemotherapy regimens to control distant metastasis is critical for improving the prognosis of DDCS. However, as mentioned above, DDCS is known to be resistant to chemotherapy. The efficacy of adjuvant chemotherapy has been reported in 23 patients out of 42 cases of DDCS [18]. The five-year survival rates of the patients treated with surgery alone and those treated with adjuvant chemotherapy were 11.8% and 4%, and the median survival time was 6.4 months and 8.4 months, respectively, so the authors concluded that adjuvant chemotherapy did not extend survival. Meanwhile, Mitchell *et al*. suggested that there was an improved survival in the DDCS patients receiving doxorubicin and cisplatin as first-line chemotherapy, although subsequent papers have failed to confirm this [10,19]. Large population clinical data to determine which regimen of chemotherapy can prolong the survival of the patients with DDCS are needed.

An abrupt interface between the low-grade cartilaginous and high-grade sarcomatous components is a critical histological feature of DDCS. In the literature, the dedifferentiated component of DDCS could be variable, including conventional osteosarcoma, malignant fibrous histiocytoma, fibrosarcoma, anaplastic spindle-cell sarcoma or telangiectatic osteosarcoma [5]. In this case, the majority of the tumor was a low-grade CS component and atypical spindle cell proliferation in a fascicular pattern with collagenous stroma, mimicking fibrosarcoma, was focally seen. Some of these cells were positive for the Ki-67 antigen, suggesting the highly proliferative nature of these cells. Recent reports have shown that only the dedifferentiated component of the DDCS may cause distant metastasis, particularly to the lungs, and metastasis-related genes including *Urokinase* and *Tissue plasminogen activator* are expressed in the metastatic lung lesions [20]. The results of molecular analyses that dissect the molecular mechanisms underlying metastasis in DDCS would therefore help to improve the future treatment of DDCS.

## Conclusion

We herein reported the first detailed case of DDCS in the cervical spine. Histologically, the low-grade chondrosarcoma component and atypical spindle cells resembling fibrosarcoma were observed in the resected tumor without a transitional zone. These features confirmed that the tumor was DDCS.

## Consent

Written informed consent was obtained from the patient for publication of this case report and any accompanying images. A copy of the written consent is available for review by the editor-in-chief of this journal.

## Abbreviations

CS: chondrosarcoma; DDCS: Dedifferentiated chondrosarcoma; CT: computed tomography; MRI: Magnetic resonance imaging; LC: local control; GyE: gray equivalent.

## Competing interests

The authors report no conflicts of interest.

## Authors’ contributions

YM, YT and KH conceived of the study, collected data and drafted the manuscript. TN and KK participated in the design of the study. SO participated in the collecting and editing of images. MH helped in drafting the manuscript. AF corrected and revised the manuscript. AS, TM, YO and YI participated in coordination and helped to draft and edit the manuscript. All authors read and approved the final manuscript.
